# Supplementary material for: Taxonomic Identification of the Arctic Strain Nocardioides Arcticus Sp. Nov. and Global Transcriptomic Analysis in Response to Hydrogen Peroxide Stress
Source: Int J Mol Sci. 2023 Sep 11;24(18):13943. doi: 10.3390/ijms241813943 (PMC10531085; doi:10.3390/ijms241813943)
Supplement: Supplementary file 1 [file ijms-24-13943-s001.zip › Table S1.pdf]

Table S1 Genes with antioxidant activity of Arc9.136 genome.

Gene	Function Description
Arc9.136_GM003016	superoxide dismutase
Arc9.136_GM000051	glutathione peroxidase
Arc9.136_GM000075	glutathione peroxidase
Arc9.136_GM000247	peroxiredoxin
Arc9.136_GM001188	peroxiredoxin
Arc9.136_GM000471	catalase-peroxidase
Arc9.136_GM001349	peroxidase
Arc9.136_GM000758	catalase
