# Supplementary material for: Taxonomic Identification of the Arctic Strain Nocardioides Arcticus Sp. Nov. and Global Transcriptomic Analysis in Response to Hydrogen Peroxide Stress
Source: Int J Mol Sci. 2023 Sep 11;24(18):13943. doi: 10.3390/ijms241813943 (PMC10531085; doi:10.3390/ijms241813943)
Supplement: Supplementary file 1 [file ijms-24-13943-s001.zip › Table S2.pdf]

Table S2 Nutrient compositions of growth media

	Ingredients (g/L)
ISP-2 medium	Malt extract 10.0; Yeast extract 4.0; D-glucose 4.0 (agar 15.0)
R2A medium	Tryptone 0.25; Casamino Acid 0.5; Yeast Extract 0.5; Starch 0.5; Dipotassium phosphate 0.3; Magnesium Sulfate 0.1; Sodium Pyruvate 0.3; Peptone 0.25; Dextrose 0.5 (ager 15.0)
Zobell 2216E medium	peptone 5.0, yeast 1.0, fltered seawa ter: ultrapure water (v/v, 2:1) (agar 15.0)
glucose asparagine medium	Dipotassium phosphate 0.5; asparagine 0.5; glucose 10.0 (agar 15.0)
