# Supplementary material for: Taxonomic Identification of the Arctic Strain Nocardioides Arcticus Sp. Nov. and Global Transcriptomic Analysis in Response to Hydrogen Peroxide Stress
Source: Int J Mol Sci. 2023 Sep 11;24(18):13943. doi: 10.3390/ijms241813943 (PMC10531085; doi:10.3390/ijms241813943)
Supplement: Supplementary file 1 [file ijms-24-13943-s001.zip › Table S3.pdf]

Table S3 Primers used in qRT-PCR

Gene	qRT-PCR primers (5'-3')	
GM000489	F：TCAACAAGAGCTTCGGTGTGGTG	R：CTTGACCAGCGTGGACTTGCC
GM000488	F：AGGACTACAAGGTCGTCGGTGAG	R：GTGTAGAGCTGCTGGAAGATCGTG
GM000764	F：GCCCTTGAACCGCTACGAGAC	R：CGATCCCGAGGTGGTAGGTGTC
GM001004	F：CGTCCAACACCTGCTTCCTCAAG	R：GATGCGGACCTCGACGTTGAC
GM002892	F：GCTGGACCACATGATCCGCATC	R：ACCTGGAAGACGCCGATGGAG
GM001599	F：GACTGCCCGATCCTCCACGTC	R：CGTAGTTGGCCGAGAGGACCAC
GAPDH	F：CCACACCGTCATCTCGAACG	R：CCCTTGACGATGCCGAACTC
