# Supplementary material for: Taxonomic Identification of the Arctic Strain Nocardioides Arcticus Sp. Nov. and Global Transcriptomic Analysis in Response to Hydrogen Peroxide Stress
Source: Int J Mol Sci. 2023 Sep 11;24(18):13943. doi: 10.3390/ijms241813943 (PMC10531085; doi:10.3390/ijms241813943)
Supplement: Supplementary file 1 [file ijms-24-13943-s001.zip › Table S4.pdf]

Table S4 Genes used in qRT-PCR

gene	foldchange	gene description
GM000489	2.63	D-xylose transport system ATP-binding protein
GM000488	2.17	D-xylose transport system substrate-binding protein
GM000764	3.35	sugar phosphate isomerase
GM001004	3.62	long-chain-fatty-acid--CoA ligase
GM002892	4.37	error-prone DNA polymerase
GM001599	3.66	DNA polymerase IV
