# Supplementary figures and images for: Taxonomic Identification of the Arctic Strain Nocardioides Arcticus Sp. Nov. and Global Transcriptomic Analysis in Response to Hydrogen Peroxide Stress
Source: Int J Mol Sci. 2023 Sep 11;24(18):13943. doi: 10.3390/ijms241813943 (PMC10531085; doi:10.3390/ijms241813943)

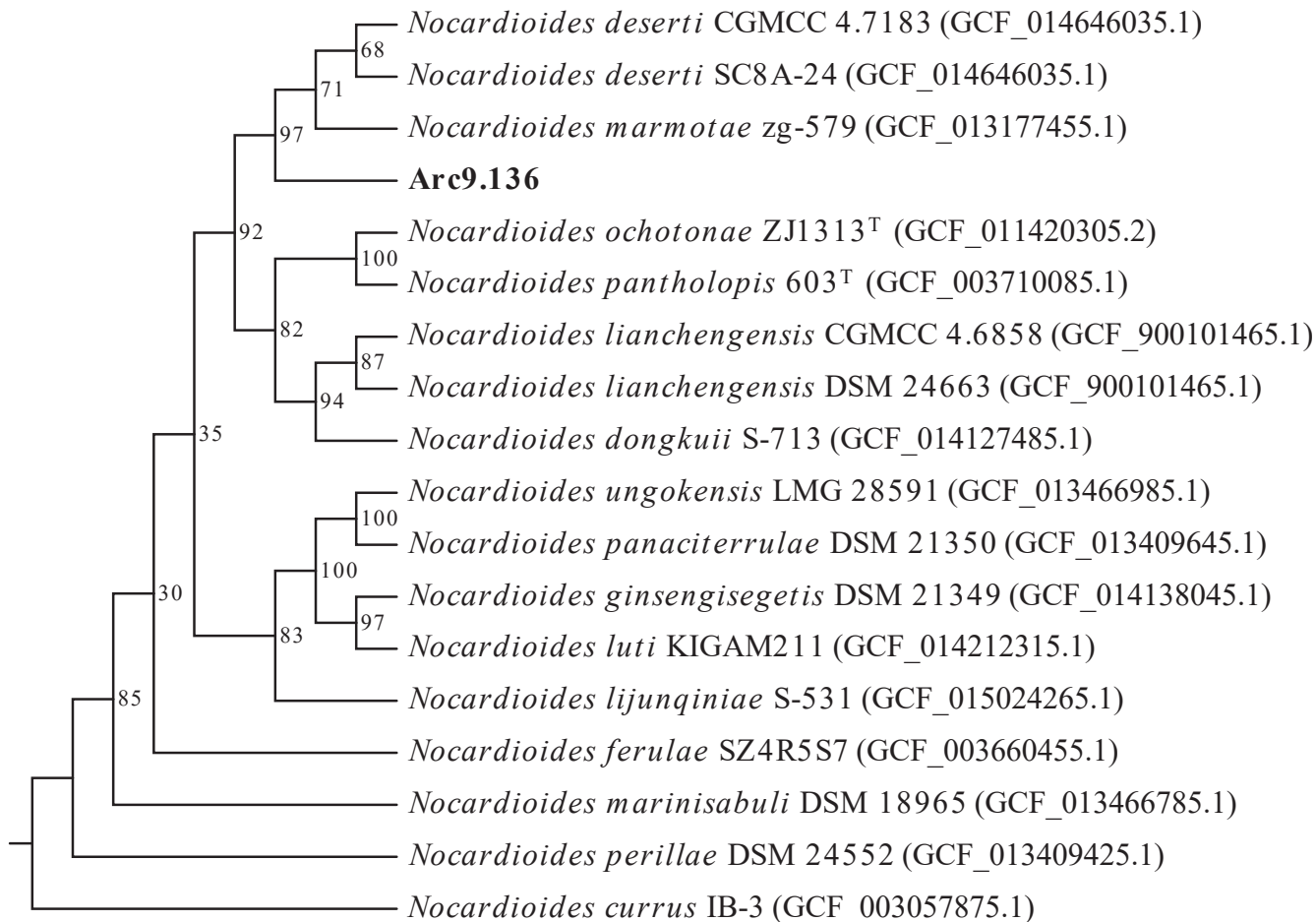

Supplement: Supplementary file 1 [file ijms-24-13943-s001.zip › Figure S1.pdf]
